# Bevacizumab Combined with Chemotherapy Improves Survival for Patients with Metastatic Colorectal Cancer: Evidence from Meta Analysis

**DOI:** 10.1371/journal.pone.0161912

**Published:** 2016-08-31

**Authors:** Irena Ilic, Slobodan Jankovic, Milena Ilic

**Affiliations:** 1 Faculty of Medical Sciences, University of Kragujevac, Kragujevac, Serbia; 2 Department of Pharmacology, Faculty of Medical Sciences, University of Kragujevac, Kragujevac, Serbia; 3 Department of Epidemiology, Faculty of Medical Sciences, University of Kragujevac, Kragujevac, Serbia; Taipei Medical University College of Medicine, TAIWAN

## Abstract

**Background:**

Colorectal cancer is one of the leading causes of cancer deaths in both sexes in the world. Improvement of existing therapy modalities and implementing new ones in order to improve survival of patients with colorectal cancer represents a great challenge for medicine. The aim of this paper was to assess the impact that adding bevacizumab to chemotherapy has on survival in patients with metastatic colorectal cancer, compared to the use of chemotherapy alone.

**Methods:**

Hazard ratios (HRs) with their 95% confidence intervals (CI) were determined from the studies and pooled. Two-sided *p* values were reported and considered to indicate statistical significance if less than 0.05.

**Results:**

A total of 12 studies that meet the inclusion criteria were identified in the literature search, 3 phase II studies and 9 phase III studies. Based on the random effects meta-analysis, a statistically significant improvement was identified for both overall survival (HR = 0.84; 95% CI: 0.74–0.94; *p* = 0.003) and progression free survival (HR = 0.64; 95% CI: 0.55–0.73; *p*<0.00001) in patients with metastatic colorectal cancer when bevacizumab was added to chemotherapy, compared to chemotherapy treatment alone.

**Conclusion:**

The findings of this meta analysis confirm the benefit of adding bevacizumab to chemotherapy in terms of survival and progression free survival, but the magnitude of this effect is not consistent throughout the included studies. This suggests the need for further research of interaction of bevacizumab with chemotherapeutic agents as well as recognition of patients’ characteristics important for the treatment selection criteria.

## Introduction

There were over 1.3 million new colorectal cancer cases discovered in 2012 based on the GLOBOCAN estimates, which makes for almost 10 per cent of all malignant tumors [1]. With about 600 000 deaths a year (8% of all deaths from malignancies), an estimated 3.5 million people in the world are living with colon cancer [1]. It is the third most common cancer in men and second in women. The 5-year survival rate for colorectal cancer is estimated to be 65% in North America, 54% in Western Europe, 34% in Central and Eastern Europe and 30% in India, China and Cuba [2]. Colorectal cancer represents a major public health issue and, bearing in mind the epidemiological data on incidence and mortality, the therapy of this disease is a great challenge for medicine. The goal of novel and future research is to improve the existing and introduce new therapeutic modalities in order to improve survival for patients with colorectal cancer.

Classification of colorectal cancer is crucial in choosing the therapy and estimating the rate of survival [3]. Surgical procedures are the main treatment in early stages. In the stage when disease has spread on the surrounding lymph nodes, a combination of anti-tumor drugs, radiation and surgical methods is applied, and in the metastatic stadium chemotherapy is administered. Standard treatment for metastatic colorectal cancer includes different chemotherapy regimens, with or without targeted molecular therapy, and surgical procedures for resectable metastases [4–6]. National Comprehensive Cancer Network’s recommendations for metastatic colorectal cancer chemotherapy include 5-flourouracil or leucovorin (5-FU/LV) with (FOLFOX) or without oxaliplatin, capecitabine with or without oxaliplatin or the use of irinotecan alone or in combination with these drugs [5].

One of the new generations of drugs in oncology, monoclonal antibodies, bind certain cell receptors through which a signal cascade is activated [4, 6, 7]. The use of bevacizumab in therapy of metastatic cancer of different localizations has led to improvement in disease outcome, which laid the groundwork for research of the effects of biological therapy in treatment of metastatic colorectal cancer [6]. In the last few years, bevacizumab, a humanized immunoglobulin G monoclonal antibody which binds to vascular endothelial growth factor, is being introduced in colon cancer treatment alongside chemotherapy [6]. Angiogenesis plays an important role in malignant tumors’ growth and persistence, and vascular endothelial growth factor is one of the central proangiogenic factors under both physiological and pathological conditions. Bevacizumab, by binding to vascular endothelial growth factor, disables its binding to vascular endothelial growth factor receptor and therefore compromises the survival of cancer cells and inhibits their growth. [7, 8]. However, while some studies have shown that the addition of bevacizumab improves survival, the results of other studies have not confirmed these findings [9].

The aim of this paper was to review the results of studies which analyzed the survival benefit that adding bevacizumab to chemotherapy has in patients with metastatic colorectal cancer compared to the use of chemotherapy alone.

## Methods

A meta analysis of the survival outcomes from studies with clinical trial design which analyzed the effects of adding bevacizumab to chemotherapy in patients with metastatic colorectal cancer was conducted.

### Search strategy

A systematic review of the literature was carried out according to a predefined protocol, in order to identify studies assessing and comparing survival of patients with colorectal cancer who are receiving chemotherapy alone or along with bevacizumab [10]. The PubMed database was searched for eligible articles (up to the end of March 2015). The following keywords were used in the search: “bevacizumab”, “metastatic colorectal cancer” and “overall survival”. Keywords were formed using the MeSH database (Medical Subject Headings Database). In case of duplicate publications, the most recent papers and those with the most data provided were selected. Finally, the “snowball” method was used, which involved tracking references and citations of found articles in order to identify additional relevant studies.

### Study eligibility criteria

Clinical trials investigating the associations between bevacizumab and overall survival in patients with metastatic colorectal cancer were initially reviewed. Two review authors (II and MI), working independently and in parallel, scanned the abstracts and then obtained and reviewed in full only studies that appeared to meet predefined inclusion and not exclusion criteria.

Inclusion criteria: clinical studies including patients with metastatic colorectal cancer, patient randomization to a group of bevacizumab + chemotherapy and chemotherapy-only group for comparison, survival as an outcome, papers in English language.

Exclusion criteria: types of paper such as meta-analyses, systematic reviews, case reports, observational studies, letters to the editor, studies that didn’t have patients assigned to a bevacizumab + chemotherapy and chemotherapy only group and didn’t measure survival as an outcome, studies not performed on humans, papers not in English language.

### Quality assessment of included studies

Methodological validity and significance was assessed for every study that met the inclusion criteria. All reviewers independently detected studies for inclusion and then separately assessed validity and significance. Any disagreements were resolved through consensus-based discussion. Quality assessment of included studies was based on the recommendations given by Higgins et al [11] and their principles for assessing risk of bias. Guidance for Assessing the Quality of Controlled Intervention Studies by National Institutes of Health [12] was also used. Methodology of studies was evaluated using available information regarding randomization, blinding, follow up–whether all patients that entered the study were properly accounted for and attributed at its conclusion, whether the groups were treated in the same way apart from the tested treatment, whether groups were similar in the beginning.

Clinical significance of included studies was evaluated by calculating Number Needed to Treat (NNT), as suggested by Altman and Andersen [13] for studies in which outcome is the time to an event. Number needed to treat was calculated using relative risk of response and absolute risk of response [14]. The NNT shows the number of patients needed to treat with the particular drug to see one additional response.

### Statistical analyses

Meta analysis was performed using the generic inverse variance method. Review Manager software Version 5.3 [15] was used. The data for ratio measures of intervention effect were entered as natural logarithms and their standard errors. The weight given to each study is the inverse of the variance of the effect estimate. Presented effect estimates were calculated using the random effects model based on the method of DerSimonian and Laird [16]. Outcome of interest, overall survival (OS), was defined as the time between randomization and any death. Progression free survival (PFS) was defined as the time between randomization and the first occurrence of progression or relapse or death from any cause. Benefit in OS and PFS from adding bevacizumab to standard chemotherapy regimens was assessed using hazard ratio (HR) and its 95% confidence intervals (CI). Data extraction was performed by directly collecting HRs from papers which reported these data. For papers that did not report HRs, software DigitizeIt was used to obtain the needed data from published Kaplan-Meier curves. These calculations were made according to the method proposed by Parmar et al [17], using the spreadsheet developed by Tierney [18]. Using the method proposed by Altman, number needed to treat to benefit and number needed to treat to harm, in terms of survival, were calculated and presented alongside their 95% confidence intervals [19].

Heterogeneity was assessed by calculating I^2^ (variation in effect size attributed to heterogeneity) as suggested by Higgins et al [20]. Clinical relevance of any heterogeneity was estimated by using tau-squared (estimate of between study variance) [21]. Results were graphically presented as forest plots with a diamond representing estimate of the pooled effect and diamond’s width representing its precision; the line of no effect is number one for binary outcomes, which, if not crossed by the diamond, depicts statistical significance. Two-sided *p* values were reported and considered to indicate statistical significance if less than 0.05.

Presence of publication bias was checked graphically using a funnel plot with effect estimate, hazard ratio, plotted on the horizontal scale and the logarithm of the standard error of hazard ratio plotted on the vertical scale.

### Ethical considerations

This study is a part of a larger research approved by the Ethics Committee of the Faculty of Medical Sciences, University of Kragujevac (Ref. No.: 01–1176).

## Results

### Literature search results

**Fig 1:**
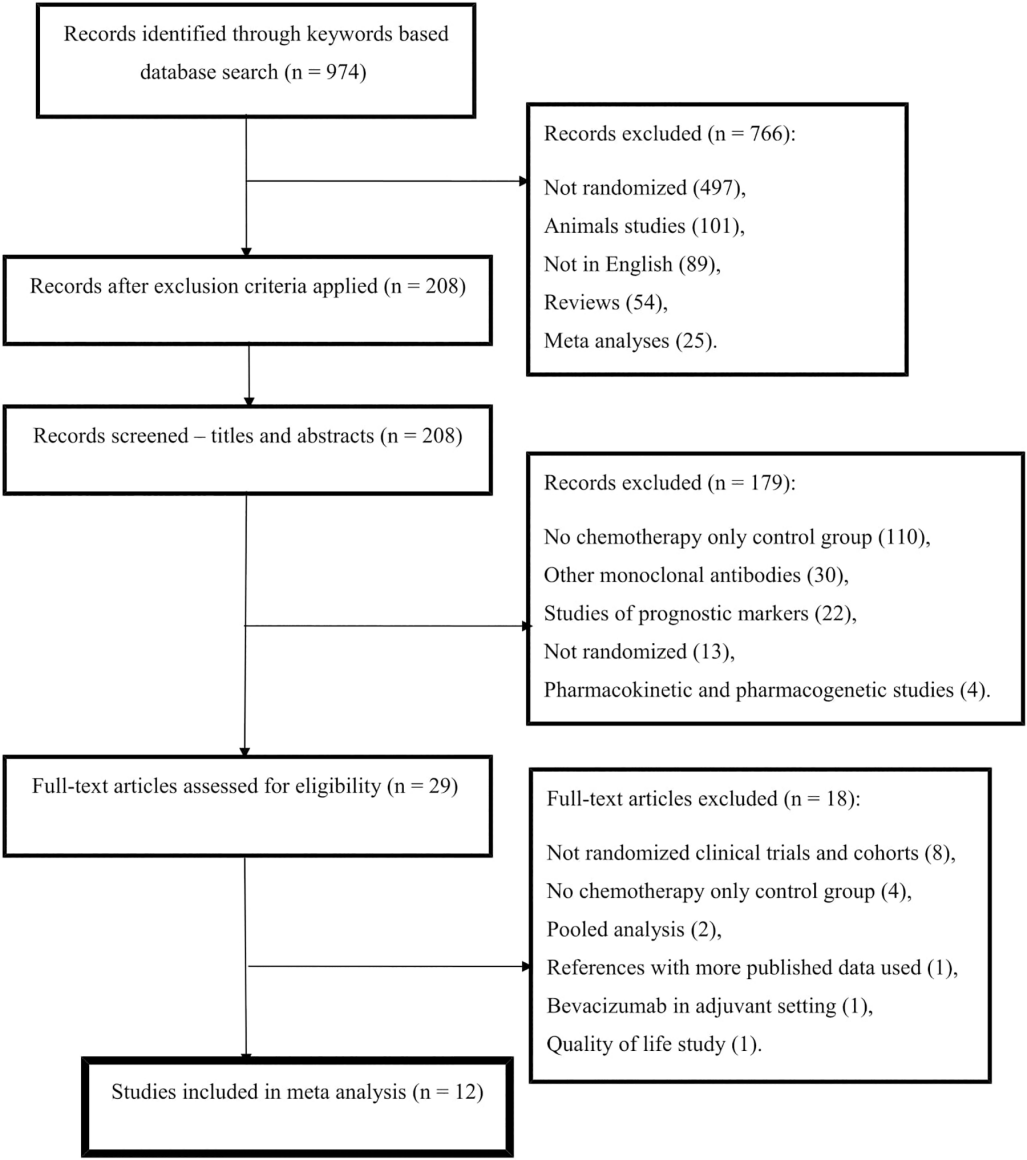
Flow diagram of study selection process.

The literature search results, based on the applied keywords and inclusion and exclusion criteria, are shown in Fig 1. This review and meta-analysis included 12 studies, totaling 5888 patients, which compared overall survival and progression free survival in patients with metastatic colorectal cancer receiving either chemotherapy alone or chemotherapy with bevacizumab. Three phase II and nine phase III clinical trials were included in our review.

### Quality assessment of included studies

Quality assessment of relevant studies was performed using the aforementioned criteria. Randomization was present and explained in detail in 10 studies, in 1 study it was stated to be present but not specified [22], and 1 study had no randomization [23] which authors admitted was a limitation. Blinding of participants and researchers was fully present in 3 studies [22, 24, 25], 2 studies didn’t state whether blinding was applied but only that an independent committee [26] and group of independent radiologists and oncologists [27, 28] had to confirm the collected data. Studies that were open-label [23, 29–33] had response to therapy assessed by an independent committee supervising the study according to Response Evaluation Criteria in Solid Tumors, or had initial data evaluation followed by a central review after which a blind clinician resolved any differences. Overall drop-out rates from the studies at endpoint were up to 20% of the number allocated to treatment. Apart from the treatment in question, other interventions were avoided or similar in the groups. Patients’ baseline demographic and clinical characteristics were well balanced throughout the studies, with authors of one study [22] overcoming gender distribution differences between study’s arms through adjustments in analysis and with one study [23] showing that differences in gender distribution were not statistically significant. Characteristics and main findings of included trials are shown in Tables 1 and 2.

**Table 1:** Characteristics of included clinical trials.

Researcher, year of publication (reference)	Study type	Number of patients	Control group	Experimental group
Kabbinavar et al., 2003 (22)	Randomized; Phase II	104	FU/LV	FU/LV+BV (5 mg/kg; 10 mg/kg)
Moehler et al., 2009 (23)	Phase II	46	CAPIRI	CAPIRI+ BV
Hurwitz et al., 2005 (24)	Randomized; Phase III	923	IFL/Placebo	FU/LV/BV
Hurwitz et al., 2004 (25)	Randomized; Phase III	813	IFL/Placebo	IFL/BV
Saltz et al., 2008 (26)	Randomized; Phase III	1401	XELOX; FOLFOX-4	XELOX+BV; FOLFOX-4+BV
Stathopoulos et al., 2010 (27)	Randomized; Phase III	222	FOLFIRI	FOLFIRI + BV
Kabbinavar et al., 2005 (28)	Randomized; Phase II	209	FU/LV/Placebo	FU/LV/BV
Guan et al., 2011 (29)	Randomized; Phase III	214	mIFL	mIFL+BV
Giantonio et al., 2007 (30)	Randomized; Phase III	829	FOLFOX4	FOLFOX4+BV; BV
Tebutt et al., 2010 (31)	Randomized; Phase III	471	Capecitabine; CM	Capecitabine + BV; CBM
Cunningham et al., 2013 (32)	Randomized; Phase III	280	Capecitabine	Capecitabine + BV
Passardi et al., 2015 (33)	Randomised; Phase III	376	FOLFIRI/FOLFOX4	FOLFIRI/FOLFOX4 + BV

Abbreviations: FU = 5-flourouracil; LV = leucovorin; BV = bevacizumab; CAPIRI = capecitabine + irinotecan; IFL = irinotecan + bolus 5-FU + leucovorin; XELOX = capecitabine + oxaliplatin; FOLFOX-4 = 5-FU + LV + oxaliplatin; mIFL = modified IFL; FOLFIRI = leucovorin + 5-FU + irinotecan; CM = capecitabine + mitomycin; CMB = capecitabine + mitomycin + B.

**Table 2:** Efficacy outcomes of included clinical trials (chemotherapy vs bevacizumab).

Researcher, year of publication (reference)	Progression-free survival (months)	Response rate (%)	Overall survival (months)
Kabbinavar et al., 2003 (22)	5.2 vs 9.0 vs 7.2	17 vs 40 vs 24	13.8 vs 21.5 vs 16.1
Moehler et al., 2009 (23)	11.4 vs 12.8	29.4 vs 34.5	15 vs 24
Hurwitz et al., 2005 (24)	6.8 vs 8.8	37 vs 40	15.1 vs 18.3
Hurwitz et al., 2004 (25)	6.2 vs 10.6	34.8 vs 44.8	15.6 vs 20.3
Saltz et al., 2008 (26)	8.0 vs 9.4	49 vs 47	19.9 vs 21.3
Stathopoulos et al., 2010 (27)		35.2 vs 36.8	22 vs 25
Kabbinavar et al., 2005 (28)	5.5 vs 9.2	15.2 vs 26	12.9 vs 16.6
Guan et al., 2011 (29)	4.2 vs 8.3	17 vs 35	13.4 vs 18.7
Giantonio et al., 2007 (30)	4.7 vs 7.3 vs 2.7	8.6 vs 22.7 vs 3.3	10.8 vs 12.9 vs 10.2
Tebutt et al., 2010 (31)	5.7 vs 8.5 vs 8.4	30.3 vs 38.1 vs 45.9	18.9 (C) vs 16.4
Cunningham et al., 2013 (32)	5.1 vs 9.1	10 vs 19	16.8 vs 20.7
Passardi et al., 2015 (32)	8.4 vs 9.6	50 vs 50.6	21.3 vs 20.8

### Number needed to treat

Number needed to treat calculated using response rates given in the included studies is shown in Table 3. This table also shows the Number needed to treat to benefit (NNTB) and Number needed to treat to harm (NNTH), in respect of survival, for addition of bevacizumab to chemotherapy for metastatic colorectal cancer. The values of number needed to treat to benefit show how many patients need to be treated with bevacizumab to see one more additional survival compared to chemotherapy-only regimens, while number needed to treat to harm shows how many patients need to be treated with bevacizumab to see one less good outcome in comparison to chemotherapy-only. These values were calculated for time points of 12 months and 24 months and presented with 95% confidence intervals which, where appropriate, included infinity, as proposed by Altman [21].

**Table 3:** Number needed to treat throughout studies.

Researcher, year of publication (reference)	Bevacizumab dose	NNT for response	NNTB and NNTH–survival	
			At 12 months	At 24 months
Kabbinavar et al., 2003 (22)	5 mg/kg; 10 mg/kg	4; 14	NNTB 9 (6–20)	NNTB 6 (5–11)
Moehler et al., 2009 (23)	7.5 mg/kg	20	NNTH 100 (NNTH 4 to ∞ to NNTB 4)	NNTB 7 (NNTB 2 to ∞ to NNTH 6)
Hurwitz et al., 2005 (24)	5 mg/kg	33	NNTB 10 (NNTB 4 to ∞ to NNTH 29)	NNTB 10 (NNTB 4 to ∞ to NNTH 34)
Hurwitz et al., 2004 (25)	5 mg/kg	10	-	-
Saltz et al., 2008 (26)	7.5 mg/kg; 5 mg/kg	RR equal (38%)	NNTB 16 (9–63)	NNTB 34 (NNTB 12 to ∞ to NNTH 43)
Stathopoulos et al., 2010 (27)	7.5 mg/kg	63	NNTH 40 (NNTH 8 to ∞ to NNTB 13)	NNTH 9 (NNTB 48 to ∞ to NNTH 4)
Kabbinavar et al., 2005 (28)	5 mg/kg	9	NNTB 10 (NNTH 31 to ∞ to NNTB 4)	NNTB 21 (NNTH 28 to ∞ to NNTB 7)
Guan et al., 2011 (29)	5 mg/kg	6	NNTB 7 (NNTB 4 to ∞ to NNTH 1250)	NNTB 34 (NNTB 6 to ∞ to NNTH 10)
Giantonio et al., 2007 (30)	10 mg/kg	7	NNTB 8 (5–21)	NNTB 14 (7–158)
Tebutt et al., 2010 (31)	7.5 mg/kg	13; 6	NNTB 15 (NNTB 6 to ∞ to NNTB 27); NNTB 1000 (NNTB 9 to ∞ to NNTH 10)	NNTB 167 (NNTB 9 to ∞ to NNTB 10); NNTH 43 (NNTH 8 to ∞ to NNTB 12)
Cunningham et al., 2013 (32)	7.5 mg/kg	11	NNTB 8 (4–53)	NNTB 10 (5–57)
Passardi et al., 2015 (33)	5 mg/kg	167	NNTH 13 (NNTH 6 to ∞ to NNTB 101)	NNTH 35 (NNTH 8 to ∞ to NNTB 14)

Abbreviations: NNT = Number needed to treat; RR = Response rate; NNTB = Number needed to treat to benefit; NNTH = Number needed to treat to harm.

### Overall survival

**Fig 2:**
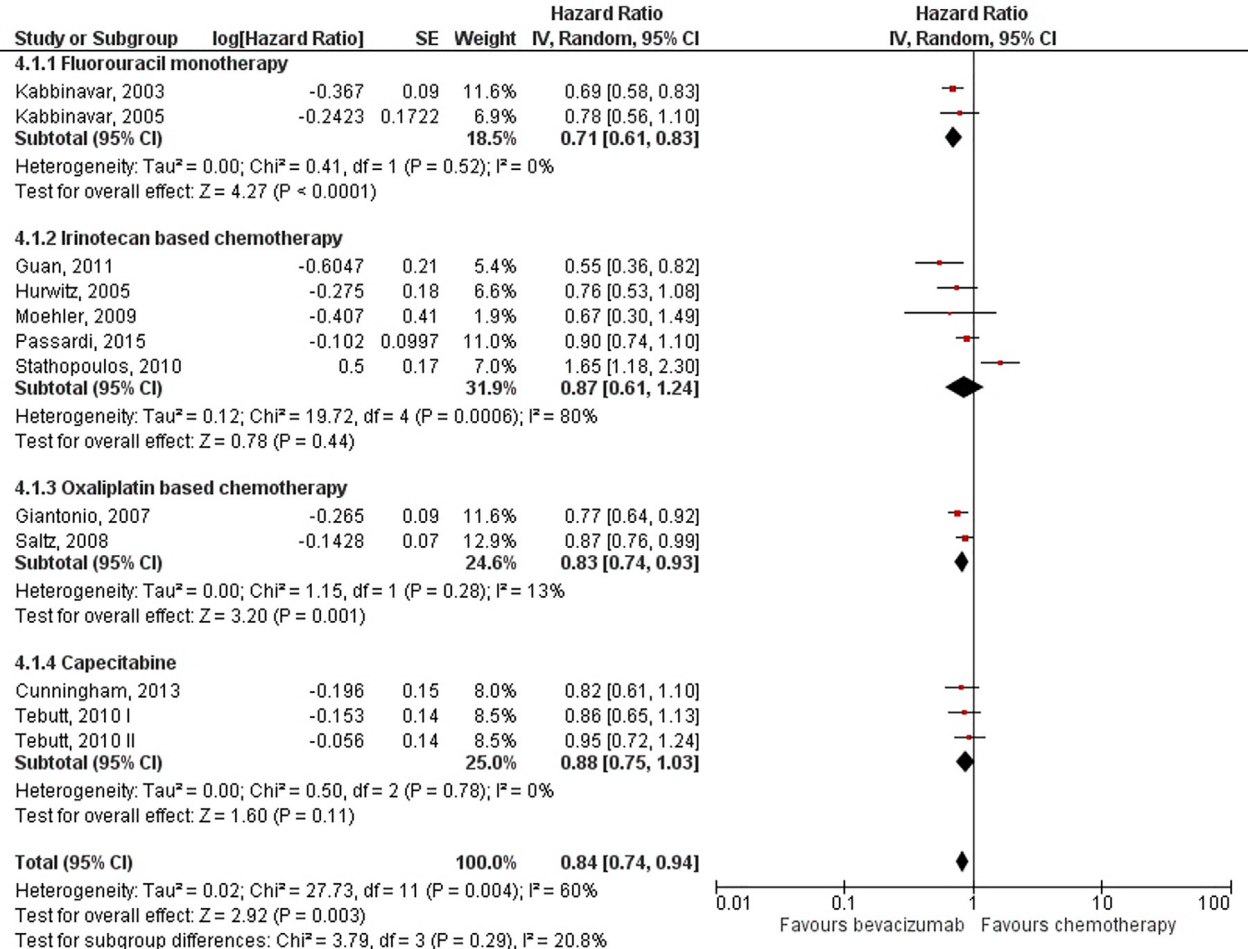
Meta-analysis of overall survival data by chemotherapy regimen. Abbreviations: SE = Standard Error; 95% CI = 95% Confidence Interval; Chi^2^ = Chi-squared test; df = degree of freedom; Tau^2^ = Tau-squared; I^2^ = I- squared; P = Probability.

**Fig 3:**
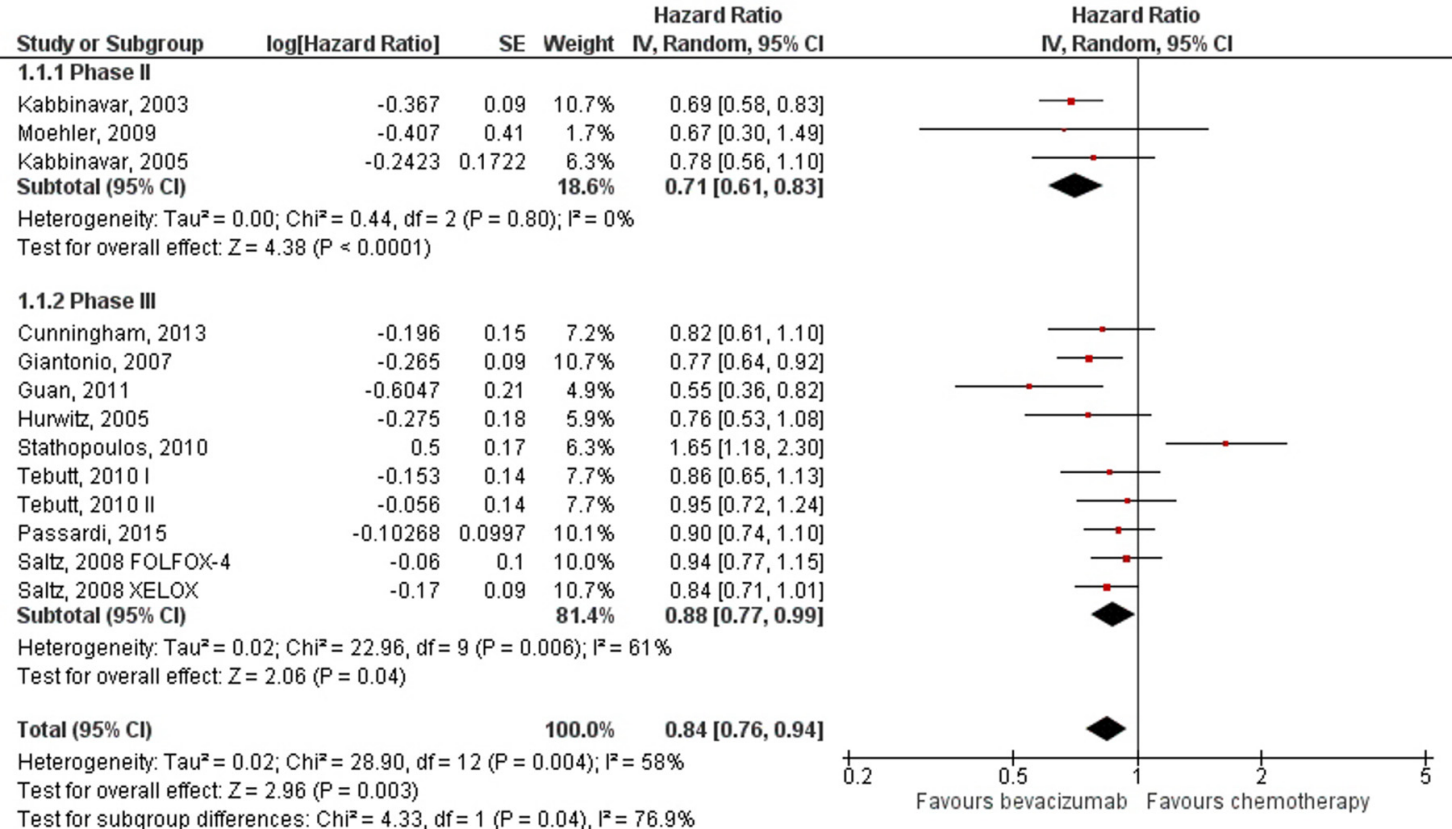
Meta-analysis of overall survival data by trial phase. Abbreviations: SE = Standard Error; 95% CI = 95% Confidence Interval; Chi^2^ = Chi-squared test; df = degree of freedom; Tau^2^ = Tau-squared; I^2^ = I- squared; P = Probability.

Addition of bevacizumab to chemotherapy showed uniform benefit regarding overall survival with HR = 0.84; 95% CI: 0.76–0.94; *p* = 0.003. Because high heterogeneity was observed between trials (I^2^ = 58%; *p* = 0.004), subgroup analyses were performed according to the chemotherapy regimen (Fig 2). Statistically significant benefit in overall survival was present in fluorouracil monotherapy (HR = 0.71; 95% CI: 0.61–0.83; *p*<0.0001) and oxaliplatin based chemotherapy regimens (HR = 0.83; 95% CI: 0.74–0.93; *p* = 0.001), while the results for irinotecan based and capecitabine regimens did not reach statistical significance. Sensitivity analysis aimed to detect the influence of three phase II studies showed results to remain stable when these studies were included or excluded from analysis (Fig 3).

### Progression free survival

**Fig 4:**
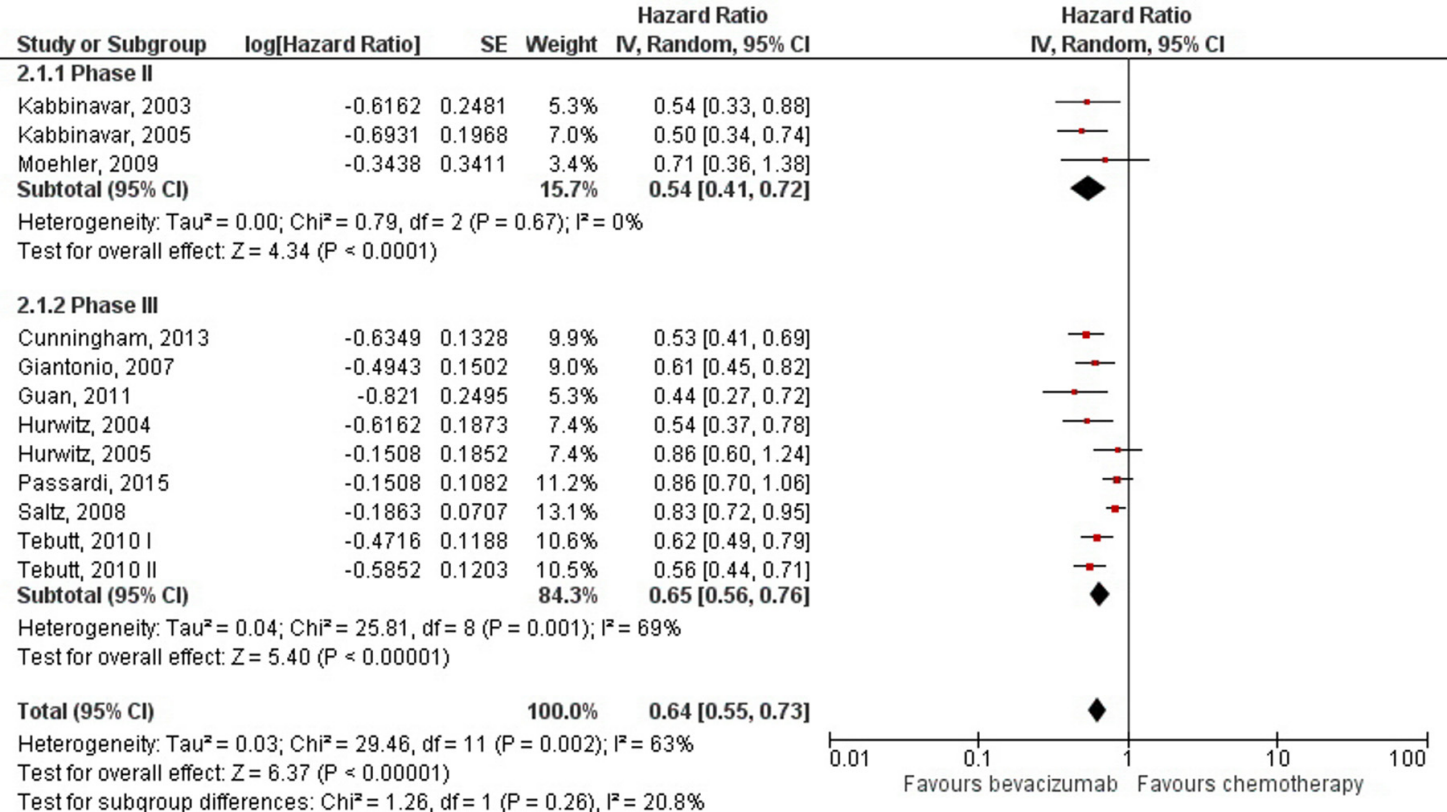
Meta-analysis of progression free survival data by trial phase. Abbreviations: SE = Standard Error; 95% CI = 95% Confidence Interval; Chi^2^ = Chi-squared test; df = degree of freedom; Tau^2^ = Tau-squared; I^2^ = I- squared; P = Probability.

**Fig 5:**
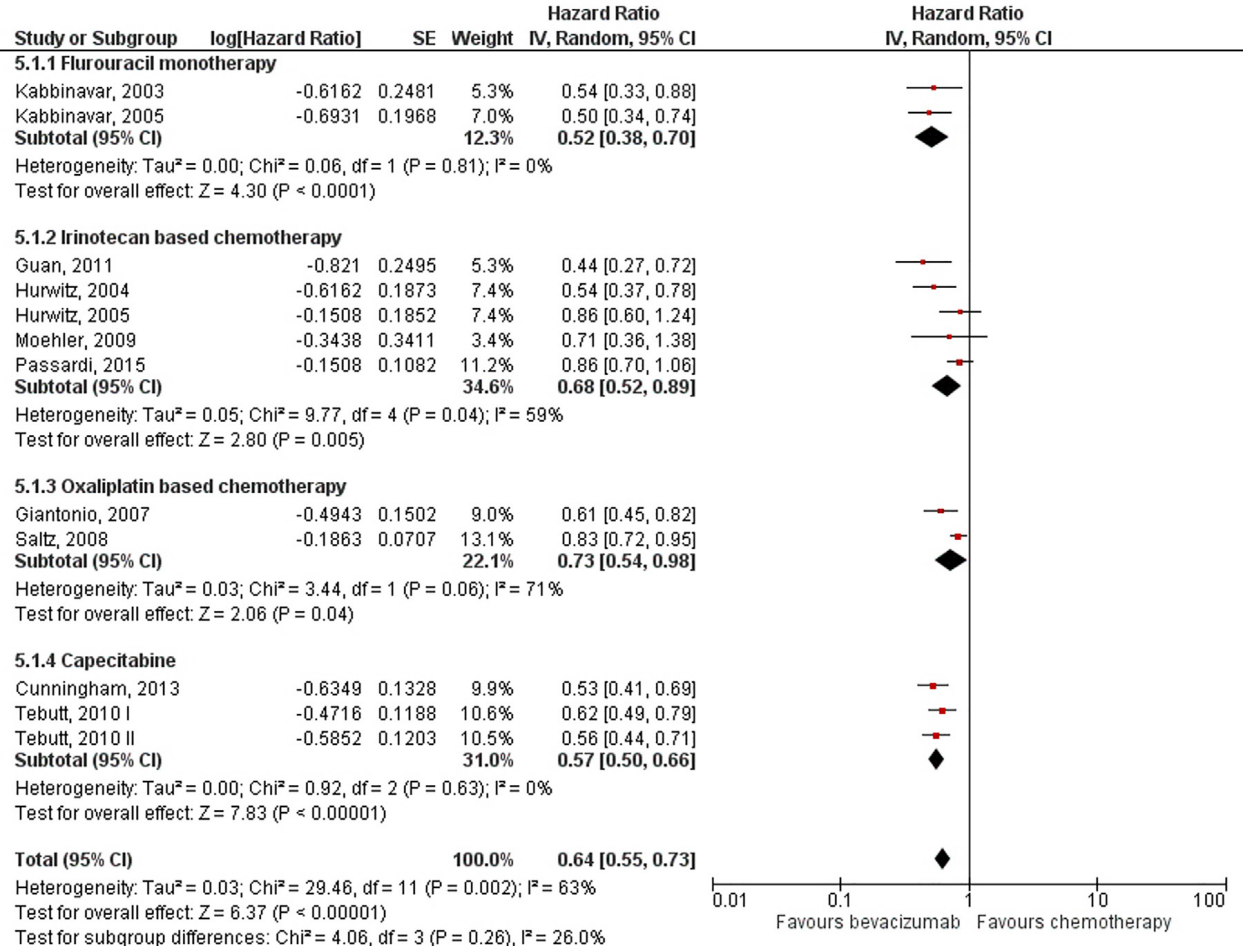
Meta-analysis of progression free survival data by chemotherapy regimen. Abbreviations: SE = Standard Error; 95% CI = 95% Confidence Interval; Chi^2^ = Chi-squared test; df = degree of freedom; Tau^2^ = Tau-squared; I^2^ = I- squared; P = Probability.

Benefit in progression free survival was evident when bevacizumab was added to chemotherapy (HR = 0.64; 95% CI: 0.55–0.73; *p*<0.00001), but with high heterogeneity (I^2^ = 63%; *p* = 0.002) (Fig 4). However, subgroup analysis showed that the increment in PFS was persistent throughout all chemotherapy regimens to which bevacizumab was added, with oxaliplatin based regimens showing the least but still significant benefit (HR = 0.73; 95% CI: 0.54–0.98; *p* = 0.04) and addition to fluorouracil monotherapy showing the most benefit (HR = 0.52; 95% CI: 0.38–0.70; *p*< 0.0001) (Fig 5).

**Fig 6:**
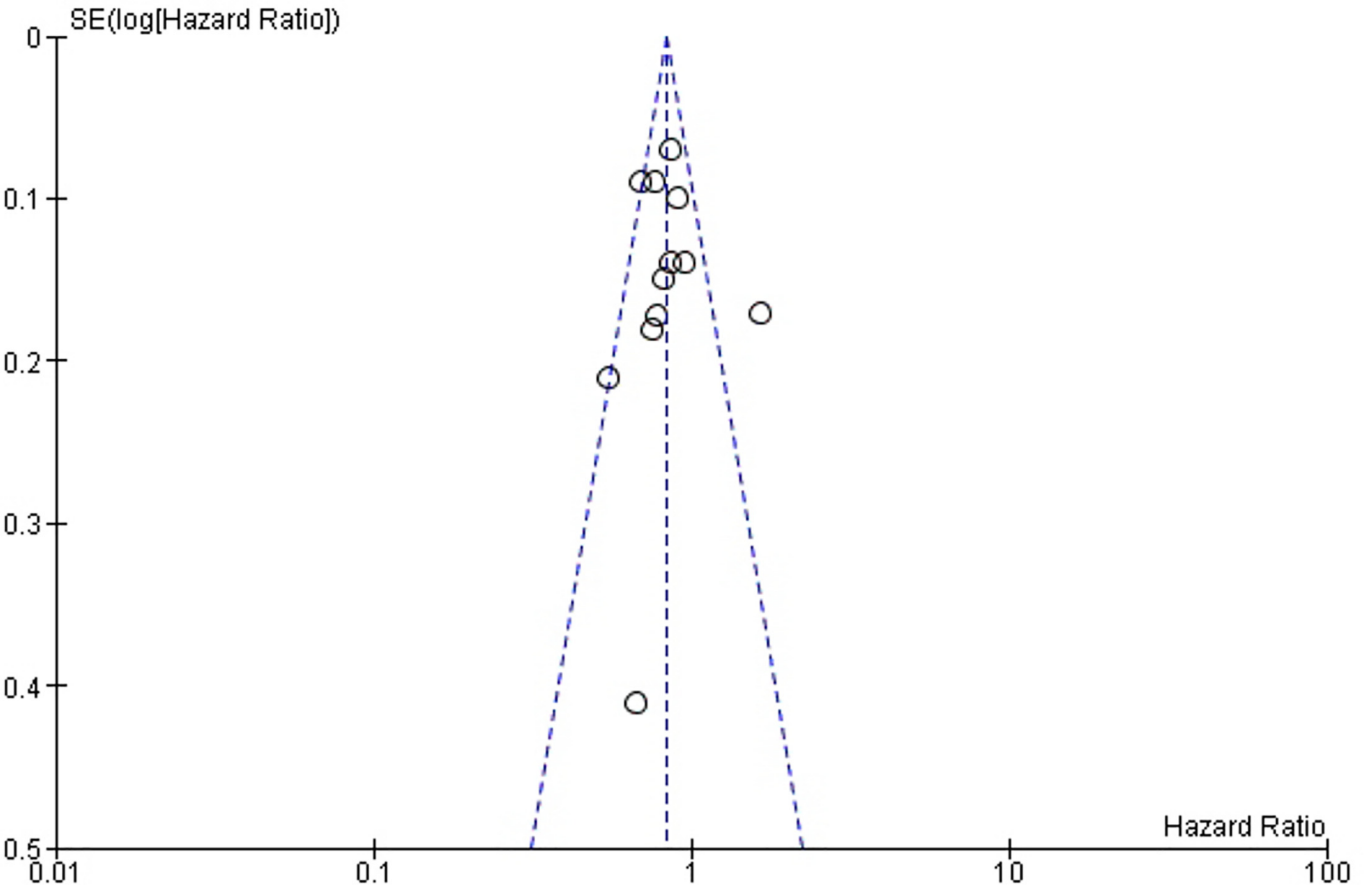
Funnel plot for publication bias assessment regarding overall survival. Abbreviation: SE = Standard Error.

**Fig 7:**
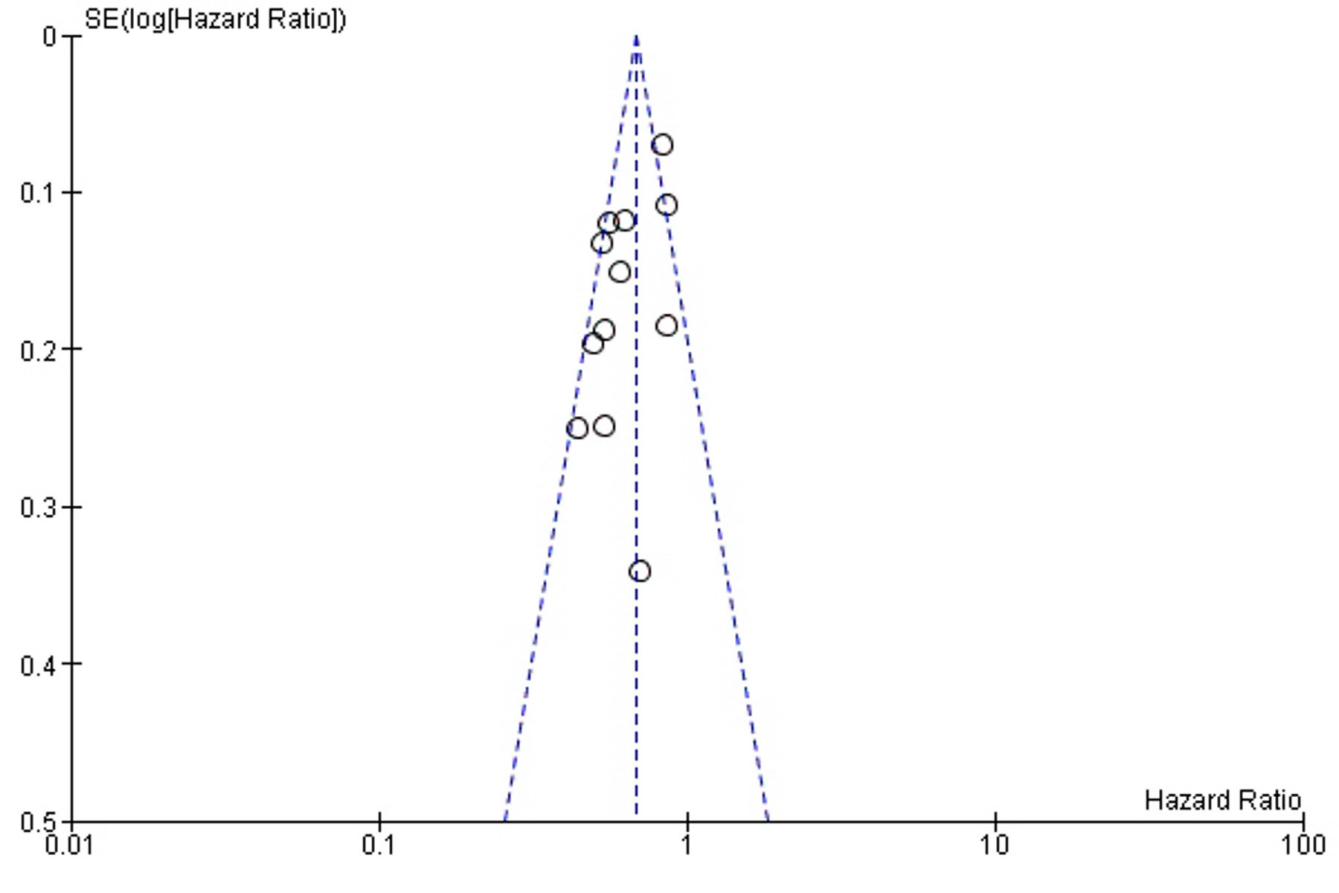
Funnel plot for publication bias assessment regarding progression-free survival. Abbreviation: SE = Standard Error.

Funnel plots (Fig 6 and Fig 7) were used for assessing publication bias. The lines representing 95% confidence intervals for each summary effect show the expected distribution of studies in the absence of selection bias or heterogeneity [34]. Funnel plots are relatively symmetrical for both OS (Fig 6) and PFS (Fig 7), and since the asymmetry is not pronounced, it is not likely that the amount of publication bias is substantial.

## Discussion

Bevacizumab is a drug that targets vascular endothelial growth factor and, as such, is widely investigated in clinical trials with cancer patients. Studies that included patients with metastatic colorectal cancer have tested it alongside different chemotherapy protocols, and this research yielded various results. These variations could find a possible explanation in studies’ settings, their methodological quality and in interactions of bevacizumab with chemotherapeutic agents. Our meta-analysis showed that the addition of bevacizumab to chemotherapy in patients with metastatic colorectal cancer prolongs overall survival and progression free survival.

Subgroup analysis by chemotherapy regimens used as the backbone of metastatic colorectal treatment shows that differences in benefit from adding bevacizumab exist. Overall survival was improved by adding bevacizumab (HR = 0.84). However, the subgroup analysis showed this effect to remain statistically significant only for fluorouracil monotherapy and oxaliplatin based regimens. When bevacizumab was added to irinotecan based regimens and capecitabine, the benefit in OS was still present but did not reach statistical significance. These findings suggest that interactions of bevacizumab with chemotherapeutic agents exist, and as such might explain the observed heterogeneity. For PFS, differences in the magnitude of bevacizumab addition effect were not as pronounced. Improvement in PFS was statistically significant (HR = 0.64), and remained so throughout different chemotherapy regimens in the subgroup analysis.

The NNT evaluated in this paper is a matter that should be further assessed. Our findings suggest that the effect of the addition of bevacizumab to chemotherapy varies among different chemotherapy regimens, different doses, but also possibly different patient’s characteristics, regarding the location of the primary tumour among others. Presence and impact of such influencing variables should be detected and weighted, which might be applicable to the selection criteria for patients in the future.

Findings of this review and meta-analysis are in concordance with those previously published. This review included new studies, detected through the search of literature based on the predefined criteria. The effect of bevacizumab on survival in patients with various cancer localizations, compared to control treatment, was assessed in meta-analyses [35,36–38] which showed that the use bevacizumab in patients with metastatic colorectal cancer was linked with a significantly greater OS and PFS. A pooled analysis of individual patient data [39] of 7 randomised clinical trials that included 3763 patients found a statistically significant increase in OS (18.7 months vs 6.4 months, *p* = 0.0003) and PFS (8.8 months versus 6.4 months, *p*<0.0001) in the group of patients who received bevacizumab in combination with chemotherapy. Authors performed a subgroup analysis according to chemotherapy protocol and confirmed these findings both for irinotecan and oxaliplatin based regimens, while our subgroup analysis did not find statistically significant benefit in OS for irinotecan based chemotherapy regimens (HR = 0.87, 95% CI 0.61–1.24; *p* = 0.44). These OS findings for the combination of bevacizumab with irinotecan based regimens are mostly influenced by one study [27] whose authors hypothesize that OS would have been greater if bevacizumab was used in maintenance treatment, because it is assumed to act as a chemotherapy-sensitizing agent. One systematic review and meta-analysis [40] evaluated the effect of adding bevacizumab to various chemotherapy protocols and found that BV significantly (*p*<0.00001) improved OS (greatest benefit found in combination with irinotecan) and PFS (greatest benefit found in combination with fluorouracil). Results of our subgroup analysis were in concordance with these regarding PFS, while for OS we found combination with fluorouracil to be the most beneficial. Authors explained that the results of their subgroup analysis for OS [40] were influenced by one single study, which might provide an explanation for these differences. Chen et al [41] found no overall survival benefit from adding bevacizumab to chemotherapy, and after performing a subgroup analysis suggested it existed only in combination with capecitabine, while they did find benefit in PFS. Reasons for these findings regarding OS might be due to insufficient data available from the ITACa trial [33] at that time, which was acknowledged by the authors [41]. Contrary to this, another meta-analysis [42] found benefit in both OS and PFS when bevacizumab was added to chemotherapy, with the subgroup analysis for OS showing it to be consistent across all chemotherapy regimens except capecitabine.

### Strenghts and limitations of this review and meta analysis

The incidence and mortality from colorectal cancer give strong implications for undertaking this review. To our knowledge, this review includes studies which are new in comparison to previously published reviews and meta analyses. In addition to presenting pooled HR for OS and PFS, we have also calculated number of patients with metastatic colorectal cancer which need to be treated with bevacizumab to gain one more response, and number needed to treat to benefit or harm, in terms of survival, alongside corresponding 95% confidence intervals. The limitation of this meta analysis is the lack of individual patient data. Also, estimating HRs from Kaplan-Meier curves, which was necessary for studies that did not report this effect measure, is another issue. Literature search which included only studies in English language was a limitation because it may have led to omitting a study published in another language.

The review of included studies showed that the addition of bevacizumab to chemotherapy in patients with metastatic colorectal cancer is associated with greater overall survival, longer progression-free survival and a better response rate compared to chemotherapy treatment alone. Although some studies showed that adding bevacizumab significantly improved survival, the results of other studies have not confirmed these findings in respect of statistical significance. Our meta-analysis combined data from 12 clinical studies and showed that adding bevacizumab to chemotherapy provides a statistically significant benefit in overall survival and progression free survival. Further investigation of survival benefit from adding bevacizumab to standard chemotherapy is needed, both in first line setting and treatment after disease progression, with emphasis on the chemotherapy protocol used as the treatment backbone. Also, studies evaluating overall survival alongside quality of life in patients with metastatic colorectal cancer are considered to be of great necessity for future research.

## Supporting Information

S1 File PRISMA Checklist. (DOC)
